# Cellular transcripts of chicken brain tissues in response to H5N1 and Newcastle disease virus infection

**DOI:** 10.1186/1743-422X-9-53

**Published:** 2012-02-23

**Authors:** Vinod RMT Balasubramaniam, Tham H Wai, Abdul R Omar, Iekhsan Othman, Sharifah S Hassan

**Affiliations:** 1 Virus-Host Interaction Group, Infectious Disease Laboratory (MR3), School of Medicine and Health Sciences, Monash University, Sunway Campus, 46150 Sunway, Malaysia; 2 Institute of Bioscience, University Putra Malaysia, UPM Serdang, Selangor 43400, Malaysia; 3 Monash University, Sunway Campus, Jalan Lagoon Selatan, 46150 Bandar Sunway, Petaling Jaya, Malaysia

**Keywords:** H5N1 Avian influenza virus, AF2240 Newcastle disease virus, mRNA differential display, Neurovirulence, Neuropathogenesis

## Abstract

**Background:**

Highly-pathogenic avian influenza (HPAI) H5N1 and Newcastle disease (ND) viruses are the two most important poultry viruses in the world, with the ability to cause classic central nervous system dysfunction in poultry and migratory birds. To elucidate the mechanisms of neurovirulence caused by these viruses, a preliminary study was design to analyze host's cellular responses during infections of these viruses.

**Methods:**

An improved mRNA differential display technique (Gene Fishing™) was undertaken to analyze differentially expressed transcripts regulated during HPAI H5N1 and velogenic neurotropic NDV infections of whole brain of chickens. The identification of differentially expressed genes (DEGs) was made possible as this technique uses annealing control primers that generate reproducible, authentic and long PCR products that are detectable on agarose gels.

**Results:**

Twenty-three genes were identified to be significantly regulated during infections with both viruses, where ten of the genes have been selected for validation using a TaqMan^® ^based real time quantitative PCR assay. Some of the identified genes demonstrated to be key factors involving the cytoskeletal system, neural signal transduction and protein folding during stress. Interestingly, Septin 5, one of the genes isolated from HPAI H5N1-infected brain tissues has been reported to participate in the pathogenic process of Parkinson's disease.

**Conclusions:**

In this limited study, the differentially expressed genes of infected brain tissues regulated by the viruses were found not to be identical, thus suggesting that their neurovirulence and neuropathogenesis may not share similar mechanisms and pathways.

## Background

Influenza is the paradigm of a viral disease in which continued evolution of the virus is of paramount importance for annual epidemics and occasional pandemics of disease in humans [1]. In particular, the Highly Pathogenic Avian Influenza (HPAI) Virus (H5N1), a member of the *Orthomyxoviridae *family of negative-stranded, segmented RNA viruses continue to pose concern both for public and animal health. As of June 22, 2011, the HPAI H5N1 avian influenza virus has caused 562 human-infected cases, and among them, 329 died [2]. The HPAI H5N1 avian influenza is also rampant in poultry with an epidemic emergence in Vietnam (2620 cases), Thailand (1140 cases), Egypt (1084 cases), 514 cases and 261 cases in Bangladesh and Indonesia respectively from the end of 2003 to 7 July 2011 [3].

Neurovirulence can be defined as the ability to undergo multicycle replication in host brain, inducing neuropathology and acute encephalitis [4,5]. The involvement of the central nervous system during influenza infections in humans and poultry is still unresolved. Although most infections of HPAI H5N1 primarily affect the respiratory system, neurological symptoms have been associated with past influenza type A outbreaks, including the 1918 "Spanish" influenza pandemic [6,7]. In a more recent note, Webster *et al*., 2004 have reported the neurovirulence of HPAI H5N1 in waterfowl and also the outbreak of HPAI in the parks of Hong Kong resulted in death of migratory birds [8]. The birds infected by H5N1 have shown acute neurological signs ranging from mild encephalitis to motor disturbances [9]. Apart from this, there are also evidence from in vitro studies showing that the infection of primary mouse microganglia and astrocyte with human H1N1 and avian H5N1 influenza viruses could possibly trigger apoptosis and also proinflammatory reactions [10,11].

The outcome of the infection is determined by complex host-virus interactions with a large number of altered transcriptional and translational rates, and functional kinetics of participating genes. There are evidences which clearly show the involvement of many host genes in innate immunity, interleukin activity and vesicle trafficking such as endocytosis and phagocytosis during virus entry in hosts infected with various types of influenza viruses [12-15]. However with regards of the neurovirulence of HPAI H5N1, the mechanism of how the virus causes the neuropathogenesis, the temporal features of the 'cross talks' between host and virus and the cellular response from the infection is still not very clear.

To better understand the molecular and cellular basis of HPAI H5N1 avian influenza virus infection and the neurovirulence caused by the virus in poultry, chicken brain tissue was chosen to be investigated in the present study. As a comparison, we also chose to investigate the mRNA transcripts regulated in brains of chicken tissue infected with Newcastle Disease Virus (NDV). NDV is an avian paramyxovirus that has recently been assigned to the new genus Avulavirus within the family Paramyxoviridae [16,17]. This particular virus causes Newcastle disease; a highly contagious and fatal viral disease affecting most species of birds especially chickens and frequently responsible for devastating losses in poultry [18,19]. NDV strains can be classified as highly virulent (velogenic), intermediate (mesogenic) or non-virulent (lentogenic). Virulent strains that cause diarrhoea and frequently haemorrhagic intestinal lesions are called viscerotropic velogenic. Strains that cause respiratory and neurotropic signs are called neurotropic velogenic [20]. We used a neurotropic velogenic strain, since chickens infected with both viruses displays similar CNS dysfunction symptoms, such as head shaking, violent tremors, and loss of balance. It would be really interesting to investigate whether both this viruses share the same mechanism or pathway in causing neurotropism towards the host.

First, the model of encephalitis of chickens infected by HPAI H5N1 and NDV was constructed, then we employed a new differential display GeneFishing™ PCR technique to compare the gene expression in normal and infected cells and tissues. This sensitive technique is based on the determination of multiple expression patterns of pre-determined sequences and we also combined it with the use of annealing control primer (ACP)™ technology in order to provide a primer with annealing specificity to the template, and allow only targeted product to be amplified without any false artifacts [21,22]. The other great advantage of this technique is that the bands can be isolated and the genes cloned in a vector for sequence identification and stored for further use. Recently, this technique was successfully used by Balasubramaniam *et al*., 2011 in comparing the mRNA transcripts regulated in 3 different host systems infected with HPAI H5N1. This study have shown host cell responses or the regulation of host cell transcriptions of different permissible cell or host systems following infection with the same virus may have their own specific patterns for altering host gene expressions and may not share similar mechanisms and pathways [23].

We hope that this present preliminary study can contribute to the comprehension of the interaction of the H5N1 and NDV virus with chickens and compare the progress of the neuropathogenesis as well as being a stepping stone for further study of interactions between the selective transcriptome which were up regulated and down regulated during the time of infection and viral proteins.

## Methods

### Viruses

Avian Influenza virus, isolate A/chicken/Malaysia/5858/2004 H5N1 was provided by Veterinary Research Institute, Ipoh, Perak, Malaysia. The viruses were initially isolated and passaged in Madin-Darby canine kidney (MDCK) cells. The virus stock was aliquoted, and titrated to determine tissue culture infection dose 50% (TCID_50_) in MDCK cells. The experiments were carried out in a Bio-safety level 3 (BSL-3) facility at the Institute of Bioscience, Universiti Putra Malaysia (UPM).

The velogenic neurotropic NDV strain AF2240 was a gift from UPM. This particular strain causes 100% mortality in susceptible flock [24]. The mean death time and intracerebral pathogenicity index of this virus was 50 hrs and 1.8 respectively, indicating its virulency. The virus was propagated in the allantoic cavities of embryonating specific pathogen free (SPF) chicken eggs. The allantoic fluid containing the virus was titrated and aliquoted in 1 ml lots and stored frozen in -20°C for further use.

### Chickens and virus infection

Twelve 4-week old SPF chickens were inoculated intranasally with 100 μL of PBS containing 10^6 ^EID_50 _virus of the HPAI H5N1 virus A/chicken/Malaysia/5858/2004 H5N1. The chickens were kept in an isolator within a BSL-3 facility of the Institute of Bioscience, Universiti Putra Malaysia, with food and water available *ad libitum*. The same batch and age-matched control chickens were treated with PBS pH 7.2. The chickens were euthanized when they displayed apparent central nerve system dysfunction symptoms or were dying. Both the control and infected chickens were sacrificed at the same time. The brain tissues were kept at -80°C until used for total mRNA extraction. This experiment was repeated, but this time using the velogenic neurotropic NDV strain AF2240. Chickens infected with the HPAI H5N1 and NDV are kept in separate isolators. All animal studies were performed according to protocols approved by Animal Ethics committee of the Institute of Bioscience, Universiti Putra Malaysia.

### Messenger RNA isolation

mRNA was extracted from the infected (HPAI H5N1 and NDV) and control whole chicken brain tissues using the RNeasy^® ^mini kit (QIAGEN Inc., Valencia, CA), according to the manufacturer's instructions.

### First strand cDNA synthesis

cDNA synthesis was carried out according to method previously described by Hwang *et al*., 2003 [21]. Briefly, the reaction was carried out by dT-ACP1, wherein the 3'-end core portion comprises a hybridizing sequence complementary to a poly A region of mRNA transcripts. Purified mRNA was incubated with 1 μL of dT-ACP1 at 80°C for 3 min, and reverse transcription was performed for 1.5 hour at 42°C in a final reaction volume of 20 μl containing 3 μg of the purified mRNA, 4 μl of 5 × reaction buffer (Promega, Madison, WI), 5 μl of deoxyribonucleotide triphosphate (each 2 mmol), 2 μl of 10 μmol cDNA synthesis primer deoxythiamine annealing control primer 1 (dT-ACP1; Table 1), 0.5 μl of RNasin^® ^RNase Inhibitor (40 U/μl; Promega), and 1 μl of Moloney murine leukemia virus reverse transcriptase (200 U/μl; Promega). First-strand cDNA was diluted by the addition of 80 μl of ultra-purified water for the GeneFishing™ PCR, and stored at -20°C until use.

**Table 1:** Primer sequence used in cDNA synthesis and ACP™-based PCR

Use	Primer name	Sequence
cDNA synthesis primer	dT-ACP1	5'-CTGTGAATGCTGCGACTACGATIIIII (T) 18-3'
Reverse primer	dT-ACP2	5'-CTGTGAATGCTGCGACTACGATIIIII (T) 15-3'
Arbitrary primer	ACP1	5'-GTCTACCAGGCATTCGCTTCATIIIIIGCCATCGACC-3'
(Forward primer)	ACP2	5'-GTCTACCAGGCATTCGCTTCATIIIIIAGGCGATGCC-3'
	ACP3	5'-GTCTACCAGGCATTCGCTTCATIIIIICCGGAGGATG-3'
	ACP4	5'-GTCTACCAGGCATTCGCTTCATIIIIIGCTGCTCGCG-3'
	ACP5	5'-GTCTACCAGGCATTCGCTTCATIIIIIAGTGCGCTCG-3'
	ACP6	5'-GTCTACCAGGCATTCGCTTCATIIIIIGGCCACATCG-3'
	ACP7	5'-GTCTACCAGGCATTCGCTTCATIIIIICTGCGGATCG-3'
	ACP8	5'-GTCTACCAGGCATTCGCTTCATIIIIIGGTCACGGAG-3'
	ACP9	5'-GTCTACCAGGCATTCGCTTCATIIIIIGATGCCGCTG-3'
	ACP10	5'-GTCTACCAGGCATTCGCTTCATIIIIITGGTCGTGCC-3'
	ACP11	5'-GTCTACCAGGCATTCGCTTCATIIIIICTGCAGGACC-3'
	ACP12	5'-GTCTACCAGGCATTCGCTTCATIIIIIACCGTGGACG-3'
	ACP13	5'-GTCTACCAGGCATTCGCTTCATIIIIIGCTTCACCGC-3'
	ACP14	5'-GTCTACCAGGCATTCGCTTCATIIIIIGCAAGTCGGC-3'
	ACP15	5'-GTCTACCAGGCATTCGCTTCATIIIIICCACCGTGTG-3'
	ACP16	5'-GTCTACCAGGCATTCGCTTCATIIIIIGTCGACGGTG-3'
	ACP17	5'-GTCTACCAGGCATTCGCTTCATIIIIICAAGCCCACG-3'
	ACP18	5'-GTCTACCAGGCATTCGCTTCATIIIIICGGAGCATCC-3'
	ACP19	5'-GTCTACCAGGCATTCGCTTCATIIIIITCTGCGAGC-3'
	ACP20	5'-GTCTACCAGGCATTCGCTTCATIIIIIGGTCACGGAG-3'

ACP, annealing control primer; the polydeoxyinosine [poly (dI)] linkers are underlined. In the table, "I" represents deoxyinosine

### Annealing control primer™-based GeneFishing™ PCR

Differentially expressed genes (DEGs) were screened by the annealing control primer (ACP) ™-based PCR method using the GeneFishing™ DEG kits (Seegene, Seoul, South Korea). The GeneFishing™ PCR technique involved an ACP™ system that had a unique tripartite structure in that its distinct 3'-end target core sequence and 5'-end nontarget universal sequence portions were separated by a regulator, it used primers that annealed specifically to the template, and it allowed only genuine products to be amplified; this process eliminates false positive results. Second-strand cDNA synthesis and subsequent PCR amplification were conducted in a single tube. Briefly, second-strand cDNA synthesis was conducted at 50°C (low stringency) during one cycle of first-stage PCR in a final reaction volume of 49.5 μl containing 3-5 μl (about 50 ng) of diluted first-strand cDNA, 5 μl of 10 × PCR buffer plus Mg (Promega, Madison, WI), 5 μl of dNTP (each 2 mM), 1 μl of 10 μM dT-ACP2, and 1 μl of 10 μM arbitrary primer preheated to 94°C (Table 1). The tube containing the reaction mixture was held at 94°C, while 0.5 μl of Taq DNA Polymerase (5 U/μl; Promega, Madison, WI) was added to the reaction mixture. The PCR protocol for second-strand synthesis was one cycle at 94°C for 1 min, followed by 50°C for 3 min, and 72°C for 1 min. After the completion of second-strand DNA synthesis, 40 cycles were performed. Each cycle involved denaturation at 94°C for 40 s, annealing at 65°C for 40 s, extension at 72°C for 40 s, and a final extension at 72°C to complete the reaction. The amplified PCR products were separated in 1.5-2% agarose gel stained with ethidium bromide [21,22].

### Cloning and sequencing

Differentially Expressed Genes (DEG) bands were extracted from gels with the QIAquick^® ^Gel extraction kit (QIAGEN Inc., Valencia, CA) and directly cloned into a TOPO-II TA cloning vector (Invitrogen, Carlsbad, CA) according to the manufacturer's instructions. The cloned plasmids were sequenced with an ABI PRISM 3100 Genetic Analyzer (Applied Biosystems, Foster City, CA) using M13 forward primer (5'-CGCCAGGGTTTTCCCAGTCAC-GA-3') or M13 reverse primer (5'-AGCGGATAACAATTTCACACAGGA-3'). All sequences were, in turn, used to perform a Basic Local Alignment Search Tool (BLAST) search for gene identification [25].

### Quantitative reverse transcription--polymerase chain reaction (qRT-PCR)

**Figure 1:**
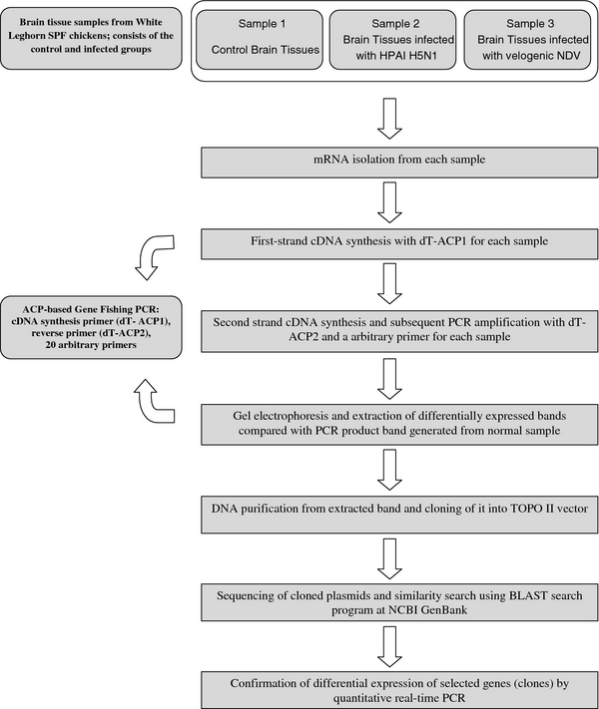
**A schematic diagram of the experimental procedure**.

For the confirmation of the differential expression of DEGs, quantitative real-time PCR was carried out for 10 DEGs selected from 23 DEGs. All RT-PCR were set up in 96-well optical plates using 50 ng of extracted uninfected and infected RNA (both H5N1 and NDV) from chicken brain tissues, 10 μl TaqMan Universal RT-PCR Master Mix (Applied Biosystems, Foster City, CA, USA), and 1 μl of primers/probe set containing 900 nM of forward and reverse primers and 300 nM probe was added to a final volume of 20 μl per reaction. All samples were tested in triplicates. RT-PCR program consisted of incubation at 48°C for 30 min, and 40 cycles at 95°C for 10 min, 95°C for 15 sec, and 60°C for 1 min with the Step One Plus Real-Time PCR System^® ^(Applied Biosystems). A non-template control and an endogenous control (eukaryotic 18 s rRNA) were used for the relative quantification. All quantitations (threshold cycle [CT] values) were normalized to that of 18 s rRNA to generate ΔCT, and the difference between the ΔCT value of the sample and that of the reference (uninfected sample) was calculated as ΔΔCT. The relative level of gene expression was expressed as 2^-ΔΔCT ^[26]. Primers for qRT-PCR were designed using Primer3 software http://frodo.wi.mit.edu/cgi-bin/primer3/primer3.cgi with these parameters: amplicon length, 95-100 bp; primer length, 18-27 nucleotides; primer melting temperature, 60-64°C; primer and amplicon GC content, 20-80%; difference in melting temperature between forward and reverse primers, 1-2°C. Primers were synthesized by Integrated DNA Technologies (Coralville, IA, USA). Primer information is listed in Table 2. The overall scheme of the experiment is shown in Figure 1.

**Table 2:** Primers used in real time PCR assays

Amplification target	Sequence (5'-3')
Hsp70 F	GGCACCATCACTGGGCTTA
Hsp70 R	TCCAAGCCATAGGCAATAGCA
Hsp70 Probe	**6FAM**-CGTGATGCGTATTATCAATGAGCCCACA-**Iowa Black FQ**
SNAP25 F	AACGAAATGGATGAGAACCTTG
SNAP25 R	TTATTGGAATCAGCCTTCTCCA
SNAP25 Probe	**6FAM**-ACGTCACATGGCCCTGGACATGGGC-**Iowa Black FQ**
CRMP2 F	AGATCACGGTGTGAATTCATTC
CRMP2 R	GGATAACACTCAGGACCTCATA
CRMP2 Probe	**6FAM**-AGACCGCTTCCAGCTGTCTGACTCGCAG-**Iowa Black FQ**
SEPT5 F	GAGAAGCAGGACCATGAGAA
SEPT5 R	GACTCTCCTGCAACCATC
SEPT5 Probe	**6FAM**-ACTCTGCCGAACCAGGTCCACCGGA-**Iowa Black FQ**
VIM F	ACAGCACATCCAAATCGATATG
VIM R	TTCGGAGAGATCTGCAAATTTG
VIM Probe	**6FAM**-ACTGCTGCCCTGCGCGATGTTCGT-**Iowa Black FQ**
TBCD F	AAACATCCTGGAGAGCTTCA
TBCD R	CTGGTACTTGTCCATTATAACGATG
TBCD Probe	**6FAM**-ACCCGGTGGCCCAAGAGGTGATCGT-**Iowa Black FQ**
Hsp60 F	CATGGTGTGACCGTAGCAA
Hsp60 R	GGCAATAGAGCGTACCAAGA
Hsp60 probe	**6FAM**-ACGAAGAGGCTGGGGATGGCACCACT-**Iowa Black FQ**
IL-8 F	CACTGTGAAAAATTCAGAAATCATTGTTA
IL-8 R	CTTCACCAAATACCTGCACAACCTTC
IL-8 probe	**6FAM**-AATGGAAACGAGGTCTGCTTAAACCCCAAG-**Iowa Black FQ**
CNTNAP5 F	GAGTTGAAATAAGATCCCCCAA
CNTNAP5 R	CATAGTGCCACTGATTGTCATT
CNTNAP Probe	**6FAM**-CGGCCCCACAGAAGCAACTGTGCAGT-**Iowa Black FQ**
TRPV6 F	CCTGTGCGTAGCGTTGGAT
TRPV6 R	GGTTCCTGCGGGTGGAA
TRPV6 Probe	**6FAM**-AACCCATCGACGAGAGGGGCCCCAT-**Iowa Black FQ**
18s rRNA F	AGCTCGTAGTTGGATTTCTGTTAATAATTTA
18s rRNA R	GCATATGCCTGCTTTAAGCACTCT
18s rRNA probe	**6FAM**-TTTCTCAAAGTAAAATTTCA-**Iowa Black FQ**

### Statistical analysis

Statistical analysis of the real-time RT-PCR data was evaluated using paired student's t test. Data were presented as mean ± SEM derived from at least three separate and independent experiments and a value of *p *< 0.05 was considered to be statistically significant.

## Results

### Cellular transcripts regulated from brain tissues infected with HPAI H5N1 and velogenic AF2240 NDV

The chickens infected with both the neurotropic viruses displayed obvious CNS dysfunction symptoms, such as head shaking, violent tremors, and loss of balance thus supporting the case of classic nonsuppurative encephalitis.

Differentially expressed mRNAs using a combination of 20 arbitrary primers and two anchored oligo (dT) primers (dT-ACP1 and dT-ACP2) of the brain tissues infected with 2 different types of viruses (HPAI H5N1 and NDV) were isolated, cloned and sequenced. For each one the brain tissue, more than 100 transcripts were observed however, only distinct up regulated or down regulated transcripts as observed on the agarose gels were chosen i.e. after exclusion of poor bands and bands which did not show much differences in intensity between the control and infected cells.

**Figure 2:**
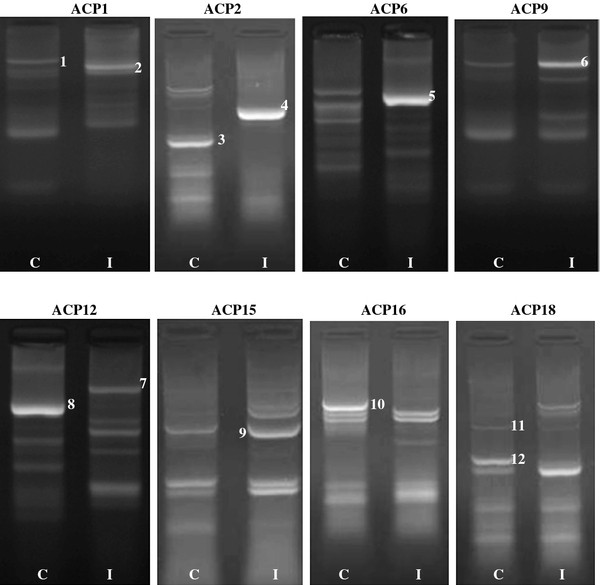
**Gel picture showing DEGs obtained from control and HPAI H5N1 infected brain tissues**. There are 12 bands, of which 6 of them are up regulated and 6 down regulated. The identities of these bands are presented in Table 3; C: Control brain tissues; I: Infected with H5N1. Each number indicates the DEG number.

**Figure 3:**
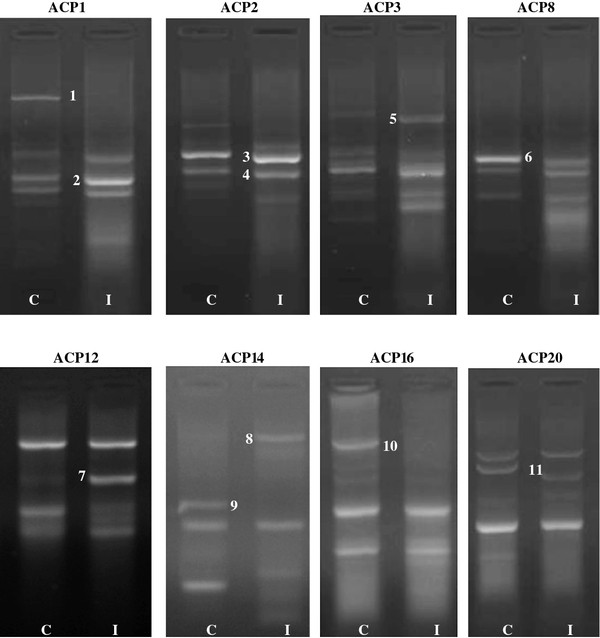
**Gel picture showing DEGs obtained from control and velogenic AF2240 NDV infected brain tissues**. There are 11 bands, of which 6 of them are up regulated and 5 down regulated. The identities of these bands are presented in Table 4; C: Control brain tissues; I: Infected with NDV. Each number indicates the DEG number.

For HPAI H5N1 infected brain (Figure 2) 12 distinct bands could be observed, of which 6 of the transcripts are up-regulated and 6 down-regulated. For NDV infected brain (Figure 3), 11 distinct bands, of which 6 are up regulated and 5 down regulated were isolated. Using the BLAST database search, the 23 differentially expressed genes were identified i.e. 12 up-regulated and 11 down-regulated genes in the samples of infected hosts, compared to the uninfected. The functional roles, sequence similarities and characterization of the differentially expressed transcripts are summarized in Tables 3 and 4. BLASTn searches in GenBank revealed that the differentially expressed genes displayed significant similarities with known genes or expressed sequence tags (ESTs).

**Table 3:** Sequence similarities and characterization of differentially expressed transcripts of HPAI H5N1 infected Chicken Brain

Clone		Accession	Homology	Putative
Name	Identity	Number	(%)	functional role
DEG 1	*Gallus gallus *vimentin (VIM), mRNA (down regulated)	NM_001048076	96%	Part of cytoskeleton system and vital in maintaining cell morphology, regulating the progress of protein synthesis, enabling cellular motion, and playing important roles in both intracellular transport and cell division.
DEG 2	*Gallus gallus *heat shock 70 kDa protein 2 (HSPA2), mRNA (up regulated)	NM_001006685	100%	Protein folding, stress response
DEG 3	Unknown sequence (down regulated)			
DEG 4	Influenza A virus (A/goose/Hubei/65/2005(H5N1)) segment 3 polymerase PA (PA) gene, complete cds (up regulated)	HM172312	99%	Subunit of the trimeric complex of the RNA-dependent-RNA polymerase of Influenza A, involved in transcription of viral RNAs
DEG 5	PREDICTED: *Gallus-gallus septin *similar to PHD finger protien 2 (LOC415981), mRNA (up regulated)	XM_414324	96%	Involved in chromatin-mediated gene regulation
DEG 6	*Gallus gallus *septin 5 (Sep-05), mRNA (up regulated)	NM_001030654	96%	parkin substrate; vesicle and membrane associated protein that plays a significant role in inhibiting exocytosis.
DEG 7	*Gallus gallus *dihydropyrimidinase-like 2 (DPYSL2), mRNA (up regulated)	NM_204494	99%	Neural signal transduction associated protein
DEG 8	PREDICTED: *Gallus gallus *hypothetical protein LOC769149 (LOC769149), mRNA (down regulated)	XM_001232361	100%	
DEG 9	*Gallus gallus *synaptosomal-associated protein, 25 kDa (SNAP25), mRNA (up regulated)	NM_205458	100%	
DEG10	***Clonorchis sinensis ***clone ACP-2U-114 (down regulated)	EU780129	100%	
DEG 11	*Gallus gallus *finished cDNA, clone ChEST766i3 (down regulated)	CR406701	96%	
DEG 12	*Gallus gallus *tubulin folding cofactor D (TBCD), mRNA (down regulated)	NM_001012801	90%	Involved in the pathway leading to correctly folded beta-tubulin from folding intermediates

**Table 4:** Sequence similarities and characterization of differentially expressed transcripts of AF2240 NDV infected Chicken Brain

Clone		Accession	Homology	Putative
Name	Identity	Number	(%)	functional role
DEG 1	PREDICTED: *Meleagris gallopavo *n-sulphoglucosamine sulphohydrolase-like (LOC100549132), partial mRNA (down regulated)	XM_003211497	99%	Involved in proteoglycan metabolic process, as well as catalytic activity, hydrolase activity, metal ion binding and sulfuric ester hydrolase activity
DEG 2	***Gallus gallus ***isolate YP20092 breed Chigulu mitochondrion, complete genome (up regulated)	GU261719	96%	
DEG 3	*Gallus gallus *interleukin 8 (IL8), mRNA (up regulated)	NM_205498	98%	Chemokine produced by macrophages and other cell types such as epithelial cells. It is one of the major mediators of the inflammatory response, particularly, serves as a chemical signal that attracts neutrophils at the site of inflammation.
DEG 4	*Gallus gallus *heat shock 60 kDa protein 1 (chaperonin) (HSPD1), nuclear gene encoding mitochondrial protein, mRNA (up regulated)	NM_001012916	99%	Protein folding, stress response.
DEG 5	*G.gallus *gene for beta-2 microglobulin (clone RG6) (up regulated)	Z48931	90%	Component of MHC class I molecules, which are present on all nucleated cells.
DEG 6	*Gallus gallus *finished cDNA, clone ChEST292n17 (down regulated)	CR391527	88%	
DEG 7	*Sus scrofa *calcium transporter 1 (TRPV6) mRNA, partial cds (up regulated)	FJ268731	90%	Member of the Transient receptor potential (TRP) family of membrane proteins, involved in Ca^2+^reabsorption.
DEG 8	*Gallus gallus *contactin associated protein-like 5 (CNTNAP5), mRNA (up regulated)	NM_001048079	96%	Member of the neurexin family which functions in the vertebrate nervous system as cell adhesion molecules and receptors play a role in the local differentiation of the axon into distinct functional subdomains.
DEG 9	*Gallus gallus *finished cDNA, clone ChEST317g8 (down regulated)	CR389719	90%	
DEG 10	Unknown sequence (down regulated)			
DEG 11	PREDICTED: *Gallus gallus *similar to CD163 molecule (LOC776417), mRNA (down regulated)	XM_001235940	99%	Member of the scavenger receptor family with cysteine-rich domains (SCRC) identified as a receptor of haptoglobin-hemoglobin (Hp-Hb) and exclusively expressed in cells of monocyte-macrophage lineage

### Transcriptional alteration of 10 selected genes of brain tissues infected with HPAI H5N1 and velogenic AF2240 NDV

**Figure 4:**
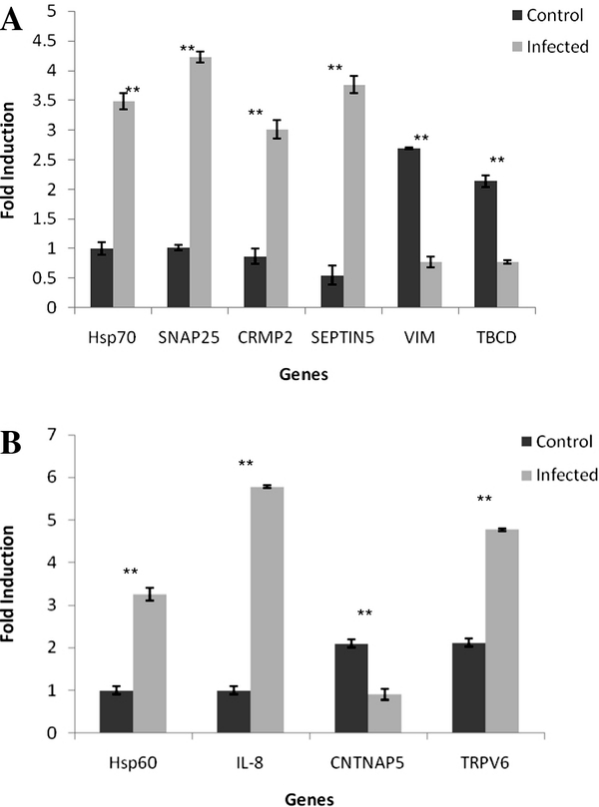
**Relative quantitation of down regulated and up regulated genes**. **A**. Relative quantitation of selected genes from control and infected with HPAI H5N1 brain tissues was determined by real time RT-PCR and the fold difference (2^-ΔΔCT^) between the study groups were calculated: Uninfected cells (Control); Infected cells (Infected). **B**. Relative quantitation of selected genes from control and infected with NDV brain tissues was determined by real time RT-PCR and the fold difference (2^-ΔΔCT^) between the study groups were calculated: Uninfected cells (Control); Infected cells (Infected). The ** (*P *value) for both sets of data is < 0.05, indicating there are significant down regulation and up regulation in the infected cells compared to control.

To confirm the efficacy of the ACP system, confirmation of the differential expression of DEGs was performed with quantitative real-time PCR for 10 DEGs selected from the total 23 DEGs using a specific primer pair for each gene (Table 5). For optimal relative quantification of the 10 selected genes, the fold difference of ΔCT (2^-ΔΔCT ± SD^) between study groups were calculated (uninfected and infected brain tissues). Resulting values were tabulated in Table 5. In general, the trends of the change of the ten genes' mRNA abundance in the infected and uninfected brain tissues were similar to the changed patterns of their chosen corresponding genes (band intensity) in agarose gels. Among these genes, in brain tissues infected with HPAI H5N1, the mRNA abundance of Hsp70, SNAP25, CRMP2, and septin5 was up-regulated, while vimentin, TBCD were down-regulated (Figure 4). Meanwhile, in brain tissues infected with velogenic neurotropic NDV strain AF2240, the mRNA abundance of Hsp60, IL-8 and TRPV6 was up-regulated while CNTNAP5 were down-regulated (Figure 4). These data provided transcriptional information complementary to the differentially expressed bands detected by the annealing control primer™-based GeneFishing™ PCR analysis.

**Table 5:** Expression changes of some of the selected genes from both the HPAI H5N1 and AF2240 NDV infected brain tissues from the qRT-PCR assay

**HPAI H5N1 infected brain tissues**		
**Gene**	**Control (2^-ΔΔCT^)**	**Infected (2^-ΔΔCT^)**
Hsp70	1.00 ± 0.11	3.48 ± 0.14
SNAP25	1.02 ± 0.05	4.23 ± 0.09
CRMP2	0.87 ± 0.13	3.01 ± 0.15
SEPT5	0.55 ± 0.16	3.76 ± 0.14
VIM	2.69 ± 0.02	0.78 ± 0.09
TBCD	2.14 ± 0.10	0.77 ± 0.03
**AF2240 NDV infected brain tissues**		
**Gene**	**Control (2^-ΔΔCT^)**	**Infected (2^-ΔΔCT^)**
Hsp60	1.00 ± 0.10	3.26 ± 0.15
IL-8	1.00 ± 0.09	5.78 ± 0.03
CNTNAP5	2.10 ± 0.10	0.91 ± 0.13
TRPV6	2.12 ± 0.10	4.77 ± 0.03

Data expressed in mean ± SEM, n = 3. Paired Student's *t*-test showed significant value of *p *< 0.05

## Discussion

Host response to viral infection involves a complex orchestration of divergent pathways designed to eliminate the virus. However, many pathways that are involved in antiviral defense can also have negative effects on the host, including cytotoxic T-lymphocyte responses, cytokine responses, and apoptosis, resulting in either dysfunction or death of infected and also neighboring uninfected cells. The potential for host pathology due to vigorous antiviral responses is of particular importance for tissues containing vital nonrenewable cell populations, such as the central nervous system (CNS) [27]. Therefore, elucidating the host response from infection of neurotropic viruses is vital in understanding the pathogenesis of CNS viral infections, particularly, in this study, both the HPAI H5N1 and the velogenic AF2240 NDV.

Gene expression has been extensively applied to screening of host cellular factors which is involved in many viral infections that may contribute to the pathogenesis of the virus. Among them are the uses of differential display in mining of host cellular genes regulated in HPAI H5N1 infection [23], microarray analysis to screen the changes of mRNA profile of mouse spleen and brain after JEV infection [28,29], and effect of rabies virus infection on gene expression in mouse brain [30]. Microarray technology permits large scale analysis of expression surveys to identify the genes that have altered expression as a result of disease. However, microarray data is known for its unreliable reproducibility of DEGs across platforms and laboratories, as well as validation problems associated with prognostic signatures [31]. In addition, identification of a gene responsible for a specialized function during a certain biological stage can be difficult to determine because the gene might be expressed at low levels, whereas the bulk of mRNA transcripts within a cell are abundant [32,33].

In this preliminary study, we utilized a new mRNA differential display GeneFishing™ PCR technique to screen for regulated cellular transcripts during HPAI H5N1 and velogenic neurotropic NDV infection in chicken brain tissue. To our knowledge, this is the first study to explore and compare the host cellular responses from these two viruses in chicken brain tissue, using this new mRNA differential display technique. This technique provides a primer with annealing specificity to the template and allows only genuine products to be amplified [21-23]. The structure of the ACP includes (i) a 3' end region with a target core nucleotide sequence that substantially complements the template nucleic acid of hybridization; (ii) a 5' end region with a non-target universal nucleotide sequence; and (iii) a polydeoxyinosine [poly (dI)] linker bridging the 3' and 5'end sequences. Because of the high annealing specificity during PCR using the ACP system, the application of the ACP to DEG identification generates reproducible, accurate, and long (100 bp to 2 kb) PCR products that are detectable on agarose gels [21-23,32,33].

The present study provides a comprehensive overview on the altered protein expression of chicken brain tissue responding to HPAI H5N1 and velogenic NDV virus infection. It proves that the function of some of the identified genes involves cytoskeleton organization, signal transduction, stress response, macromolecular biosynthesis, and metabolism. Apart from this, the present study also identified the HPAI H5N1 PA gene transcript in the infected chicken brain tissue (Figure 2 and Table 3), demonstrating that the brain tissues were infected by the virus.

Overall, our studies have demonstrated that the host cellular responses of the brain tissues to infections with the HPAI H5N1 and velogenic NDV were substantially different from each other. The functional roles, sequence similarities and characterization of differentially expressed transcripts are summarized in Tables 3 and 4. Some of the selected identified differential transcripts participating in different biological progresses were discussed as follows.

Chicken Vimentin was found to be down regulated in brain during infections with HPAI H5N1. Vimentin shows the most complex pattern of expression of all known intermediate filament (IF) proteins [34] in the host cytoskeleton system. The cytoskeleton system is vital in maintaining cell morphology, regulating the progress of protein synthesis, enabling cellular motion, and playing important roles in both intracellular transport and cell division. The obtained data have strongly indicated the important role of the cytoskeleton system in the progress of HPAI infection in chicken brain tissue as vimentin was significantly down regulated after infection. Apart from that, recent evidence demonstrates that various viruses manipulate and utilize the host cytoskeleton to promote viral infection [35]. Several studies have shown that human immunodeficiency virus type 1 protease cleaves the IF vimentin and induces the collapse of vimentin in infected cells [36,37]. Immunofluorescence assay study also have clearly demonstrated that the vimentin and beta-tubulin networks collapse and disperse in IBDV-infected cells, prompting speculations to be made whether cytoskeletal disruption may be a critical mechanism of IBDV particle release from infected cells [35,38]. Further elucidation is required to determine whether HPAI H5N1 uses a human immunodeficiency virus-like strategy to cleave vimentin, resulting in highly decreased expression and the collapse of the vimentin network to release viral particles.

We also found both the stress response proteins, Hsp60 and Hsp70 are significantly up regulated in both the velogenic NDV and HPAI H5N1 infection in chicken brain respectively. HSP70 is one of the major classes of chaperone molecules and plays many roles in eukaryotic cells, especially when the host is under stress from abiotic factors such as heat and also pathogen attack (plant journal). However, cumulative findings indicate that the induction of Hsp70 is not a general response to viral infection but, instead, a highly specific response with regard to both the infecting virus and the host cell. Phillips *et al*., 1991 [39] conducted an experiment on the induction of the 70-kDa family of heat shock genes in monkey and human cells infected with different DNA viruses, such as adenovirus type 5, herpes simplex virus type 1, simian virus 40, and vaccinia virus. It appeared that only adenovirus type 5 and herpes simplex virus type 1 were able to induce Hsp70 and that, between three examined Hsps, only Hsp70 was induced, thus accounting for a highly specific response. Apart from, this, there is evidence showing the constitutive form Hsc70 as a target protein of the multistep process required for the entry of rotavirus into epithelial intestinal cells [40,41]. On the other hand, Hsp60 is another type of stress protein that have important role in correct folding of proteins and stabilizing unfolded labile proteins [42]. The heat shock response is a homeostatic mechanism that that protects cell from damage by up regulating the expression of genes that code for Hsp60 [43]. The up regulation of Hsp60 production allows for the maintenance of other cellular processes occurring in the cell, especially during stressful times. However, Sung and Guhung, 2001 have also shown that Hsp60 is an essential factor for the activation of human Hepatitis B Virus polymerase for it to function inside cellular environment, proving the direct involvement of this protein in viral pathogenesis [44]. It would be really interesting to investigate further whether Hsp70 and Hsp60 have similar role in the pathogenesis of HPAI H5N1 and velogenic NDV in brain.

From this study also, we have substantial amount of data to suggest that the HPAI H5N1 disrupts normal neural signal transduction associated transcripts in brain. We found that, CRMP2, Septin 5 and SNAP25 genes were significantly up regulated in chicken brain in the time of infection. The chicken collapsin (formerly known as semaphorin) response mediator protein (CRMP)-62 molecule (also known as CRMP2) was originally identified as a signalling molecule of Sema3A, a type of semaphorin protein. The semaphorins constitute a major family of axon guidance cues in central as well as peripheral nervous system [45]. Uchida *et al*., 2005 have demonstrated that sequential phosphorylation of CRMP2 by cyclin-dependent kinase 5 (cdk5, a proline-directed serine/threonine kinase) and GSK3β was a vital progress of Sema3A signaling [46]. On the other hand, Gao *et al*., 2001 have evidence showing Cdk5 mediates changes in morphology and promotes apoptosis of astrocytoma cells [47]. So, we would like to postulate that the activation of CRMP2 by cdk5 could be related to the neuron cells apoptosis in chicken brain tissues, induced by HPAI H5N1. Apart from this, Septin5 was also significantly up regulated during infection with HPAI H5N1. Septin5, a parkin substrate, is a vesicle and membrane associated protein that plays a significant role in inhibiting exocytosis. Studies have shown that homologues of Septin5, such as SEPT5 v1 and SEPT5 v2a can bind and be ubiquitinated by parkin. The levels of both these septins can also be modulated by parkin. Both SEPT5 v1 and SEPT5 v2 accumulate in brains of patients with ARJP, whereby patients with ARJP show increased levels of SEPT5 v1 and SEPT5 v2 compared to controls suggesting that parkin is essential for the normal metabolism of these proteins. Unfortunately, insufficient knowledge is available to determine how elevated levels of SEPT5 v1 and SEPT5 v2 might contribute to the pathophysiology of ARJP [48]. Further studies needs to be conducted to elucidate the role played by septins in neurovirulence of HPAI H5N1. Another important finding in this study is the up regulation of SNAP25 transcript in brain tissues infected with HPAI H5N1, a gene involved in synaptic physiology of the brain. SNAP25 gene gives rise to proteins which are known as SNAREs. These particular groups of proteins are involved in all fusion competent membranes of the cell [49]. Fusion cannot occur without SNAREs and is blocked when SNAREs are removed genetically or destroyed by neurotoxins [50,51]. SNAP25 are involved in docking of synaptic vesicles [52] to the presynaptic membrane and thus play a key role in the release of neurotransmitters at the synapse [53], interestingly found to be down regulated in brain tissues infected with rabies virus, resulting in reduced release and uptake of neurotransmitters [54,55], contradicting with our findings.

CD163, a member of the scavenger receptor family with cysteine-rich domains (SCRC) identified as a receptor of haptoglobin-hemoglobin (Hp-Hb) and exclusively expressed in cells of monocyte-macrophage lineage was significantly down regulated in brain tissues infected with velogenic NDV. CD163 has been identified as a marker for perivascular macrophages in humans, monkeys, and mice. There are also evidence showing that perivascular CD163 expression is up regulated and the number of CD163-positive cells increases in HIV and SIV encephalitis (HIVE and SIVE) brains [56,57]. However, CD163 is not a "classical" activation marker, because peripheral blood monocytes and most tissues macrophages of normal uninfected controls all express it and because in vitro pro-inflammatory stimuli largely down-regulate its expression, thus suggesting that the down regulated transcript in velogenic NDV infected brain tissue is due to decreased number of activated perivascular macrophages resulted by inflammatory disorder-related apoptosis response.

## Conclusions

Conclusively, the chickens infected by the HPAI H5N1 A/chicken/Malaysia/5858/2004 H5N1 and velogenic neurotropic NDV strain AF2240 displayed severe CNS dysfunction, and infected chickens developed classic nonsuppurative encephalitis. We have managed to identify and isolate 23 authentic genes which were up and down regulated in this study. Some of the identified genes demonstrated to be key factors in the cytoskeletal system, neural signal transduction and protein folding during stress. Interestingly, one of the identified genes, Septin5, participated in pathogenic process of Parkinson's disease which also developed encephalitis and CNS dysfunction as well. Although, the current data is just a preliminary one, it may suggest that both the HPAI H5N1 and velogenic neurotropic NDV may have different mechanism in causing neurovirulence towards the host. Further elucidation of these genes is needed to facilitate the understanding the mechanism of neurovirulence of both viruses especially the interaction of viral proteins with the identified genes.

## Abbreviations

HPAI: Highly pathogenic avian influenza; NDV: Newcastle disease virus; CNS: Central nervous system; ACP: Annealing control primer; DEG: Differentially expressed genes; TCID: Tissue culture infective dose; EID: Egg infective dose; SPF: Specific pathogen free; PBS: Phosphate buffered saline; Hsp60: Heat shock protein 60; Hsp70: Heat shock protein 70; SNAP25: Synaptosomal-associated protein of 25 kDa; CRMP2: Collapsin response mediator protein 2; SEPT5: Septin 5; VIM: Vimentin; B2M: Beta-2 microglobulin; TRPV6: Sus scrofa calcium transporter 1; JEV: Japanese encephalitis virus; IF: Intermediate filament; cdk5: Cyclin-dependent kinase 5; ARJP: Autosomal recessive juvenile parkinsonism; SNARE: Soluble NSF(N-ethylmaleimide-sensitive factor) attachment protein receptor.

## Competing interests

The authors declare that they have no competing interests.

## Authors' contributions

SSH and VRMTB conceived of the study, participated in its design and coordination. VRMTB and THW performed most of the experiments, VRMTB prepared the manuscript while SSH, ARO, and IO discussed the results and revised the manuscript. All authors have read and approved the final manuscript.
